# Thymosin β_4_ and β_10_ Levels in Pre-Term Newborn Oral Cavity and Foetal Salivary Glands Evidence a Switch of Secretion during Foetal Development

**DOI:** 10.1371/journal.pone.0005109

**Published:** 2009-04-01

**Authors:** Sonia Nemolato, Irene Messana, Tiziana Cabras, Barbara Manconi, Rosanna Inzitari, Chiara Fanali, Giovanni Vento, Chiara Tirone, Costantino Romagnoli, Alessandro Riva, Daniela Fanni, Eliana Di Felice, Gavino Faa, Massimo Castagnola

**Affiliations:** 1 Dipartimento di Citomorfologia, Università di Cagliari, Cagliari, Italy; 2 Dipartimento di Scienze Applicate ai Biosistemi, Università di Cagliari, Cagliari, Italy; 3 Istituto di Biochimica e di Biochimica Clinica, Università Cattolica and/or Istituto per la Chimica del Riconoscimento Molecolare, CNR, Istituto Scientifico Internazionale (ISI) Paolo VI, Roma, Italy; 4 Istituto di Clinica Pediatrica, Divisione di Neonatologia, Università Cattolica, Roma, Italy; University of Giessen Lung Center, Germany

## Abstract

**Background:**

Thymosin β_4_, its sulfoxide, and thymosin β_10_ were detected in whole saliva of human pre-term newborns by reversed-phase high performance chromatography coupled to electrospray ion-trap mass spectrometry.

**Methodology/Principal Findings:**

Despite high inter-individual variability, concentration of β-thymosins increases with an inversely proportional trend to postmenstrual age (PMA: gestational age plus chronological age after birth) reaching a value more than twenty times higher than in adult whole saliva at 190 days (27 weeks) of PMA (thymosin β_4_ concentration: more than 2.0 µmol/L *versus* 0.1 µmol/L). On the other hand, the ratio between thymosin β_4_ and thymosin β_10_ exhibits a constant value of about 4 along all the range of PMA (190–550 days of PMA) examined. In order to investigate thymosin β_4_ origin and to better establish the trend of its production as a function of gestational age (GA), immunohistochemical analysis of major and minor salivary glands of different pre-term fetuses were carried out, starting from 84 days (12 weeks) of gestational age. Reactive granules were seen in all glands with a maximum of expression around 140–150 days of GA, even though with high inter- and intra-individual variability. In infants and adults reactive granules in acinar cells were not observed, but just a diffuse cytoplasmatic staining in ductal cells.

**Significance:**

This study outlines for the first time that salivary glands during foetal life express and secrete peptides such as β-thymosins probably involved in the development of the oral cavity and its annexes. The secretion increases from about 12 weeks till to about 21 weeks of GA, subsequently it decreases, almost disappearing in the period of expected date of delivery, when the gland switches towards the secretion of adult specific salivary peptides. The switch observed may be an example of further secretion switches involving other exocrine and endocrine glands during foetal development.

## Introduction

Beta thymosins are ubiquitous peptides having interesting intra- and extra-cellular functions, whose name derives from their first characterization from thymus extracts [1], [2]. Thymosin β_4_ (Tβ_4_) is usually the most abundant β-thymosin in the cytoplasm of most cell types and it plays pivotal roles in the cell cytoskeletal system as G-actin sequestring peptide [3]. Tβ_4_ frequently co-exists with a second member of the β-thymosin family, i.e. thymosin β_10_ (Tβ_10_) in man, cat, rat, mouse and rabbit, thymosin β_9_ in sheep, calf and pig [4]. Although the secretion pathway is not fully understood, recent studies highlighted various extra-cellular roles for these peptides [1]. For instance, Tβ_4_ modifies the rate of attachment and spreading of endothelial cells on matrix components inducing matrix metalloproteinases [5], it stimulates the migration of human umbilical vein endothelial cells [6], it is involved in the repair of damaged cornea [7], it induces hair growth by activating hair follicle stem cells [8], and it might be involved in the development and repair of heart [9] and brain damages [10]. Whereas Tβ_4_ is a potent enhancer of angiogenesis, Tβ_10_ inhibits it and changes of the two peptides ratio can exert either positive or negative control [11]. Tβ_4_ has been detected in human whole saliva and tears by immunological techniques [12] and recent studies of our group evidenced that in the oral cavity a main contribution derives from crevicular fluid [13], where, as demonstrated by Reti and co-workers [14], Tβ_4_ plays an important role in suppressing the production of interleukin-8 following stimulation by tumour-necrosis factor α and it acts on the whole as antimicrobial, anti-inflammatory and anti-apoptotic peptide on gingival fibroblasts. This study describes the detection of high concentrations of thymosins β_4_ and β_10_ in the whole saliva of pre-term newborns, during a survey carried out by proteomic approaches. Because a crevicular origin for these peptides in newborns has to be obviously excluded, further immunohistochemical analyses of the major and minor salivary glands of human fetuses and newborns of different postmenstrual age (PMA: gestational age (GA) plus chronological age after birth) were performed, in order to establish the source of the high amounts of Tβ_4_ detected in whole saliva and to add further information on potential roles of this peptide during foetal development.

## Materials and Methods

### Ethics statements

The study protocol and written consent forms were approved by the Pediatric Department Ethics Committee of the Faculty of Medicine of the Catholic University of Rome (according to the instructions of the Declaration of Helsinki). Full written consent forms were obtained from the parents of the newborns and all rules were respected.

### Reagents

All common chemicals and reagents were of analytical grade and were purchased from Farmitalia-Carlo Erba, (Milan, Italy), Merck (Damstadt, Germany) and Sigma Aldrich (St. Louis, MI, USA). Standards of thymosin β_4_ and thymosin β_10_ were purchased from Bachem (Bubendorf, Switzerland).

### Apparatus

The HPLC-ESI-IT-MS apparatus was a Surveyor HPLC system (ThermoFinnigan, San Jose, CA, USA) connected by a T splitter to a PDA diode-array detector and to an LCQ Deca XP Plus mass spectrometer. The mass spectrometer was equipped with an electrospray ion (ESI) source. The chromatographic column was a Vydac (Hesperia, CA, USA) C8 column, with 5 µm particle diameter (column dimensions 150×2.1 mm).

### Subjects enrolled

Twelve newborns with a birth weight ranging between 500 g and 1250 g and gestational age between 27–31 weeks (193–217 days), admitted to the Neonatal Intensive Care Unit (NICU) were enrolled for this study. Infants with major congenital malformations or prenatal infections were excluded. Relevant clinical information and major clinical outcomes with regard to the study group are provided in our previous studies [15].

Moreover, 4 at-term infants (2 females, 2 males) born after uncomplicated pregnancies and vaginal delivery and admitted to Policlinico “A. Gemelli” nursery, were studied. Their mean±SD GA and birth weight were 272±7 days (38±1 weeks) and 3280±150 grams, respectively. All these 4 newborns had no clinical problems and therefore they were discharged after three days and breast-fed.

### Sample collection and treatment

Whole saliva samples were collected from pre-term and at-term newborns with a very soft plastic aspirator during the usual newborn management. Pre-term newborn sample collection was performed during several weeks after birth at established time intervals (one or two weeks). When possible, it was also performed after discharge from the neonatal unit, during the periodical check visits with about one year follow-up. Saliva was not collected from newborns when sample collection seemed to cause even a minimal stress to the newborn and/or to his/her parents. For these reasons, sample collection was more frequent during the hospital stay and sporadic after this period. After collection samples were immediately mixed with an equal volume of 0.2% 2,2,2 trifluoacetic acid (TFA) (v/v) in ice bath. After stirring, the acidic solution was centrifuged at 9000 g for three min to remove the precipitate (mainly mucins) and the acidic solution was immediately analyzed by HPLC-ESI-MS (100 µL, corresponding to 50 µL of whole saliva) or stored at −80°C until the analysis.

### RP-HPLC-ESI-MS analysis

The following solutions were utilized for the chromatographic separation: (eluent A) 0.056% aqueous TFA and (eluent B) 0.050% TFA in acetonitrile-water 80/20 (v/v). The gradient applied was linear from 0 to 55% in 40 min, at a flow rate of 0.30 mL/min. The T splitter addressed a flow-rate of about 0.20 mL/min towards the diode array detector and 0.10 mL/min towards the ESI source. During the first 5 min of separation the eluate was not addressed to the mass spectrometer to avoid instrument damage due to the high salt concentration. The diode array detector was set at a wavelength of 214 and 276 nm. Mass spectra were collected every 3 millisecond in the positive ion mode. MS spray voltage was 4.50 kV and the capillary temperature was 220°C.

### Detection of β-thymosins in whole saliva and determination of their concentration

Deconvolution of averaged ESI mass spectra was automatically performed either by the software provided with the Deca-XP instrument (Bioworks Browser) or by MagTran 1.0 [16] software. Experimental Mass values were compared with average theoretical values available at the Swiss-Prot data bank (http://us.expasy.org/tools), where Tβ_4_ and Tβ_10_ have the P62328 and P63313 codes, respectively. Identification of thymosin β_4_ and thymosin β_10_ was based on the coincidence of elution times, ESI-MS spectra and ESI-MS/MS spectra with peptide standards, as described in previous studies [13]. Thymosin β_4_ sulfoxide was identified by the coincidence of elution times, ESI-MS and ESI-MS/MS spectra with a standard obtained by treating thymosin β_4_ with H_2_O_2_. Under the chromatographic conditions used the elution times measured at the peak maximum were: Tβ_4_, 20.0 (±0.4) min; Tβ_4_ sulfoxide, 18.5 (±0.4) min; and Tβ_10_, 21.3 (±0.4) min. The average masses obtained after deconvolution of the ESI spectra were: Tβ_4_ 4963.5±0.4, Tβ_4_ sulfoxide 4979.5±0.4, Tβ_10_ 4936.5±0.4 Da.

The concentration of β-thymosins was determined by considering the extracted ion current (XIC) peak area, which is linearly related to the peptide absolute quantity, as established performing LC-MS analyses on β-thymosin standards. The linear correlation between absolute amount of the standards and XIC peak area (r = 0.999) provided the following relationship: 1 picomol = 2.1×10^6^ (XIC peak area) for Tβ_4_, Tβ_10_ and Tβ_4_ sulfoxide. The concentration was therefore computed by considering the absolute quantity and injected sample volume (usually 100 µL of acidic solution corresponding to 50 µL of WS). The XIC peaks were revealed by selecting the following charged ions: Tβ_4_, [M+5H]^5+^ = 993.8 m/z; [M+4H]^4+^ = 1241.9 m/z; [M+3H]^3+^ = 1655.5 m/z; Tβ_4_ sulfoxide [M+5H]^5+^ = 996.9 m/z; [M+4H]^4+^ = 1245.9 m/z; [M+3H]^3+^ = 1660.8 m/z; Tβ_10_, [M+5H]^5+^ = 988.3 m/z; [M+4H]^4+^ = 1235.1 m/z; [M+3H]^3+^ = 1646.5 m/z. The window for all these values was ±0.5 m/z. The percentage error of the measurements was less than 10%.

### Immunohistochemical analyses

Samples of the parotid, submandibular and minor salivary glands (the posterior superficial lingual glands or Weber) at the base of the tongue were obtained at autopsy from 5 subjects: two foetuses of 12 and 13 weeks of gestational age, respectively, and 3 pre-term infants, with a gestational age of 20, 21 and 31 weeks. In addition, two specimens of the major sublingual gland, the only two that we were able to obtain with certainty from this gland, belonging to the pre-term infants with 21 and 31 weeks of gestational age, were studied. Moreover, specimens of only submandibular and sublingual glands were obtained at the autopsy from a sixth subject, an 18 months old boy who underwent a sudden death. As a control group we utilized surrounding tissue of surgical specimens from the peritumoral parotid gland of 5 adult patients who underwent surgery for resection of pleomorphic adenoma. Tissue samples were formalin-fixed, paraffin embedded and routinely processed. Deparaffinized 5 µ-thick sections were immunostained with the antibody directed against Tβ_4_ (rabbit) at a dilution of 1∶500. The polyclonal antibody from Bachem-Peninsula Lab (San Carlos, CA, USA) has not cross reactivity against Tβ_10_ and Tα_1_. The staining procedure was ensured by using a biotin-labeled affinity isolated goat anti-rabbit IgG followed by streptavidin conjugated to horseradish peroxidase (Dako, Cytomation LS-AB2 System – HRP, Denmark). Sections of reactive lymph nodes rich in macrophages (positive controls) and lymphocytes (negative controls) were also used.

## Results

### Determination of β-thymosin concentrations in different samples under analysis

In general, the study of Tβ_4_ and Tβ_10_ concentrations in the saliva during development of human newborns evidenced a marked inter-individual variability and an overall age-dependent trend, showing decrease of thymosin ‘s concentration as a function of PMA.

**Figure 1:**
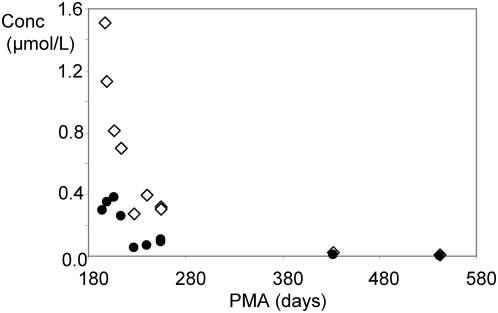
Decrease of the concentration of thymosin β_4_ (white symbols) and thymosin β_10_ (black symbols) in whole saliva as a function of postmenstrual age (days) of a pre-term newborn.

**Figure 2:**
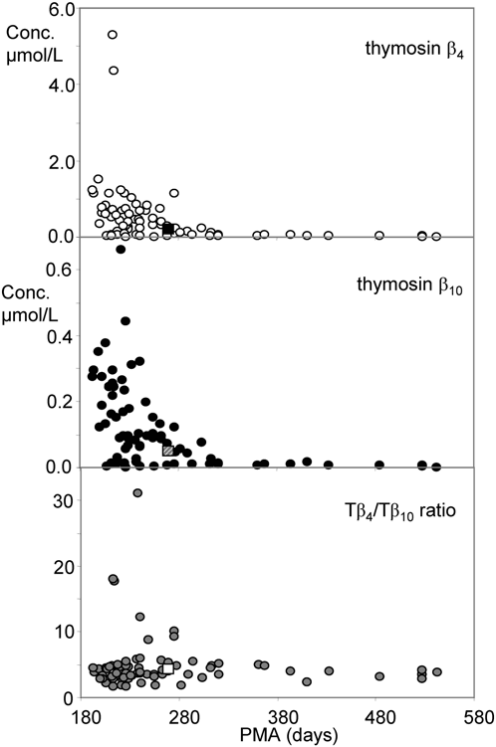
Concentration of thymosin β_4_ (white symbols, upper graph) and thymosin β_10_ (black symbols, middle graph) in whole saliva of pre-term newborns (12 subjects, 78 measurements) as a function of postmenstrual age (days). In the lower graph the Thymosin β_4_/Thymosin β_10_ ratio is reported. The square symbols in the three graphs correspond to the mean values determined in at-term newborns (4 subjects).

Figure 1 shows the concentration of Tβ_4_ and Tβ_10_ detected in whole saliva of one pre-term infant plotted as a function of postmenstrual age, for example. Both thymosins β_4_ and β_10_ show high concentration values immediately after birth at 26–27 weeks (193 days of GA, in this subject Tβ_4_ concentration in the range 1.0–1.5 µmol/L) which constantly decrease as a function of PMA, reaching values comparable to that ones of adult (Tβ_4_ concentration about 0.1 µmol/L [13]) after 38 weeks (270 days) of PMA. Similar age-dependent trends were observed for all the investigated pre-term newborns despite the high inter-individual variability, as evident from Fig 2 (12 pre-term newborns, 78 measurements at different days of PMA). In the majority of subjects the concentration of Tβ_4_ measured in the range 27–31 weeks (190–210 days) of PMA was 1–2 µmol/L, but one subject showed a spike to about 6 µmol/L. The concentration of Tβ_10_ in the same range of PMA was between 0.1–0.4 µmol/L, with one subject with a spike of 0.6 µmol/L (but not the same showing theTβ_4_ spike). In Fig. 2 are also reported (squares) the mean values observed in four at-term newborns (270 days of gestational age, 38 weeks) which values for Tβ_4_ were 0.21±0.12 µmol/L and 0.049±0.022 µmol/L for Tβ_10_. Fig. 2 also shows the ratio between the two β-thymosins concentrations in the different samples under analysis, which has a constant value of about 4 in all the range of PMA studied (at-term newborns comprised), except sporadic increases in some pre-term newborns not connected to any evident clinical outcome. Obviously, the constant ratio implies that the concentration of the two peptides correlates with high statistical significance (N = 78, r = 0.679, p<0.001).

**Figure 3:**
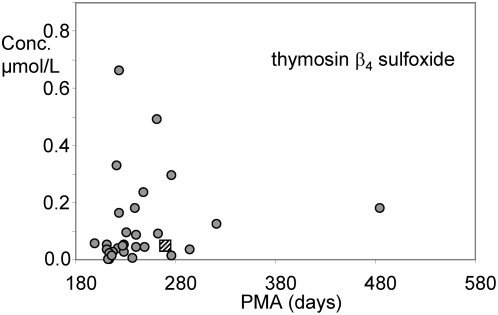
Concentration of thymosin β_4_ sulfoxide in whole saliva of pre-term newborns as a function of postmenstrual age (days). This derivative of thymosin β_4_ was detected only in 27 whole saliva samples (out of the 78 samples of Fig. 2). The square symbol corresponds to the mean value determined in at-term newborns (2 out of 4 subjects).

Similarly to the spikes of Tβ_4_/Tβ_10_ concentration ratio, no clinical outcome can be associated until now to the presence of Tβ_4_ sulfoxide sporadically detected in whole saliva of some pre-term newborns as shown in Fig. 3 (27 out of 78 measurements). The oxidation of Tβ_4_ methionine provides a sensible decrease of the interaction with G-actin [17]. Some subjects did not show the presence of Tβ_4_ sulfoxide in any sample of whole saliva collected at different days of PMA, others showed its presence immediately after birth in the period 190–230 days of PMA, others showed its presence two or three weeks after birth corresponding to 230–260 days of PMA, while Tβ_4_ sulfoxide was almost completely absent in whole saliva of newborns after 270 days of PMA and it was detected only in two out of four at-term newborns, with a mean value of 0.042 µmol/L (square of Fig. 3). In any case, when present, its mean percentage value was about 10% and it never overcame 25% of total Tβ_4_.

Because the main origin of Tβ_4_ and Tβ_10_ in adult oral fluid is the crevicular fluid [13] and this origin is not possible in newborn saliva, we were induced to establish by immunohistochemistry, thanks to the availability of specific antibodies, the source responsible for the high levels of Tβ_4_ detected by HPLC-MS analysis in the oral cavity of newborns.

### Histology and immunohistochemistry

Immunoreactivity for Tβ_4_ was detected in all cases tested in this study, both in major and minor salivary glands obtained from foetuses, newborns and adult patients. We observed marked inter-individual differences both in the degree of reactivity and in the immunohistochemical pattern. Moreover, a striking variability in the expression and in the localization of the peptide was observed among the different salivary glands tested, even in the same subject. As for morphological observations, the salivary gland structure before birth showed different degrees of immaturity, typical of differentiation from tubular structures towards acinar and ductal cells.

**Figure 4:**
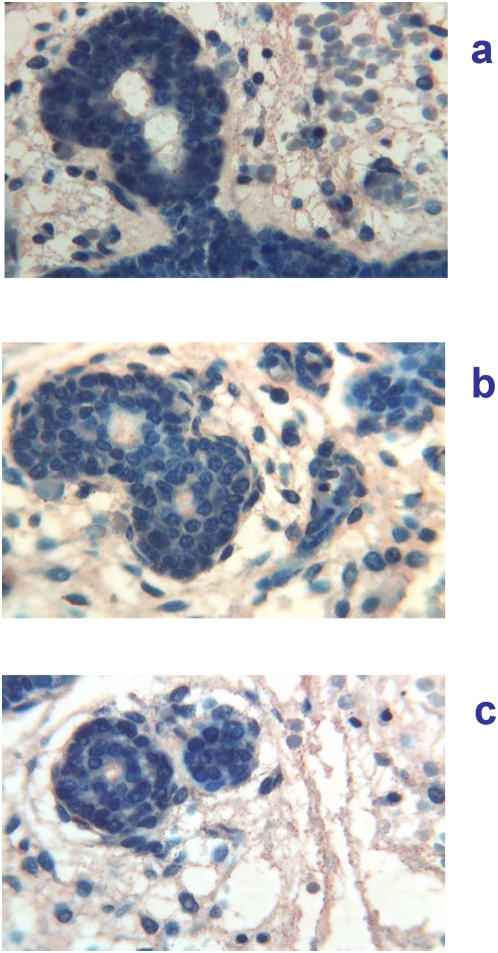
Immunohistochemical detection of thymosin β_4_ in salivary glands at 12 weeks (84 days) of gestational age. a) (400×) Parotid gland showing a granular immunoreactivity for Tβ_4_ in the cytoplasm of tubular cells and particularly at the luminar membrane and inside the lumen. Particulate deposits of the peptide are also detected in the mesenchyma surrounding the epithelial structures. b) (400×) Submandibular glands: a minor expression of the peptide is detected in the cytoplasm of tubular cells and inside the lumen. Granular deposits are also present in the mesenchyma. c) (400×) Minor salivary glands: it is possible to detect fine particulate deposits of Tβ_4_ in the tubular epithelium and in the surrounding mesenchyma.

12 weeks of gestation (84 days of GA). At this gestational age, major salivary glands showed marked immaturity, typical of the tubular phase [18]. In the parotid gland a particulate immunoreactivity for Tβ_4_ was found in the cytoplasm of tubular cells and particularly at the internal site of the luminar membrane and inside the lumen. Particulate deposits of the peptide were also seen in the mesenchyma surrounding epithelial cells (Fig 4a). A minor expression of the peptide was observed in the other major salivary gland tested (Fig 4b). Minor salivary glands at the base of the tongue showed an immature architecture, characterized by branching solid cell nests and tubuli dispersed in abundant loose mesenchyma. Fine particulate deposits of Tβ_4_ are detectable at this magnification in the tubular epithelium and in their lumen (Fig 4c).

**Figure 5:**
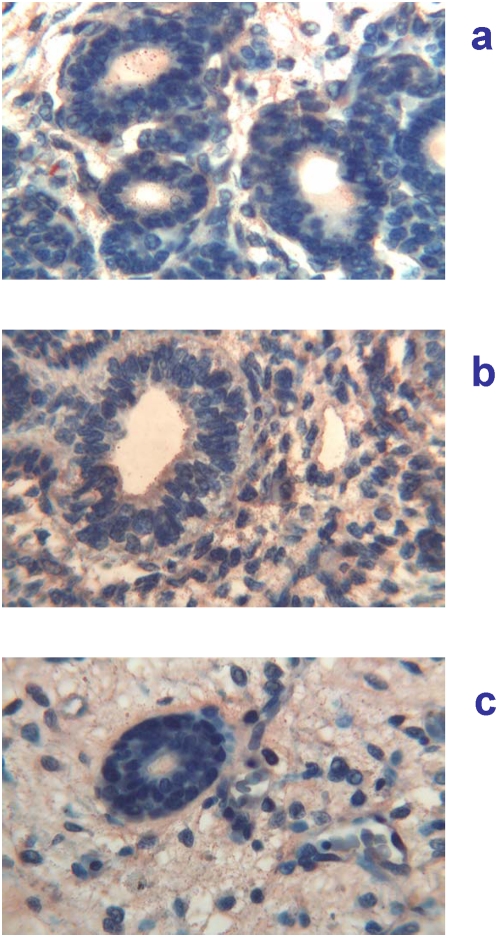
Immunohistochemical detection of thymosin β_4_ in salivary glands at 13 weeks (91 days) of gestational age. a) (400×) abundant coarse granular deposits of Tβ_4_ are observed inside the lumen of tubular structures and in the apices of tubular cells of parotid gland. b) (400×) submandibular glands showing a granular reactivity for Tβ_4_ in the apices of tubular cells and, occasionally, within the lumen. Abundant reactive granules are also observed dispersed in the mesenchyma. c) (400×) A very mild immunoreactivity for the peptide is present in immature minor salivary gland.

13 weeks of gestation (91 days of GA). The expression of Tβ_4_ was similar to that observed in the previous stages, but for the marked differences in the degree of positivity among the major and minor salivary glands. The highest reactivity for Tβ_4_ was observed in parotid glands (Fig 5a) and particularly in the tubular lumen; that seen in submandibular glands was somewhat lesser (Fig 5b); an even lower degree of reactivity was seen in minor salivary glands (Fig 5c).

**Figure 6:**
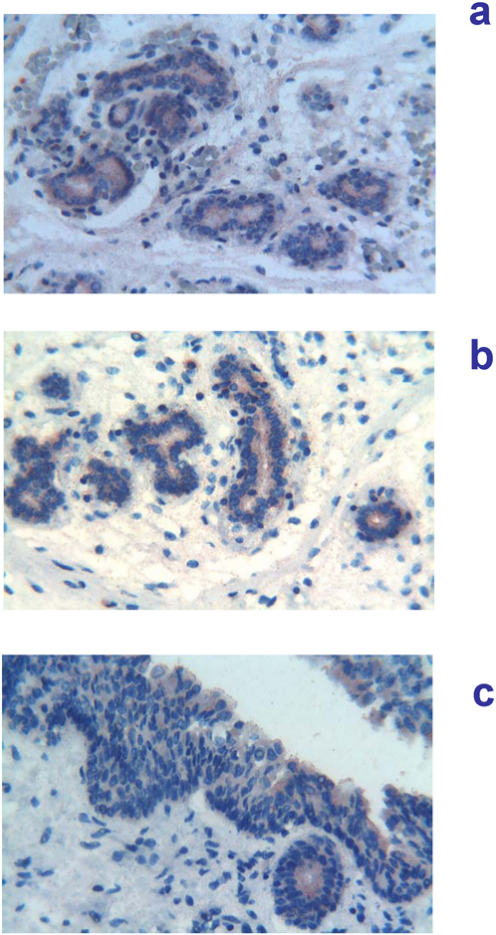
Immunohistochemical detection of thymosin β_4_ in salivary glands at 20 weeks (140 days) of gestational age. a) (250×) Parotid gland: Tβ_4_, is stored in punctate granules dispersed inside the cytoplasm of immature duct cells, particularly in the apices. Some immunoreactivity for the peptide is also present in the loose mesenchyma. b) (250×) Submandibular glands: immature tubular structures are strongly positive for Tβ_4_ , which is stored in punctate granules dispersed inside the cytoplasm of immature cells. c) (250×) Immature minor salivary glands containing few reactive granular deposits in the apices of epithelial cells, in the absence of a significant positivity in the loose mesenchyma.

20 weeks of gestation (140 days of GA). The parotid glands appeared immature, with branching structures surrounded by abundant loose connective tissue. The epithelium was strongly positive for Tβ_4_, which was stored in punctate granules dispersed inside the cytoplasm of immature cells and, in higher amounts, in the apices and within the lumen (Fig. 6a). Some immunoreactivity was also present in the mesenchymal space, in between the glandular structures. Occasionally, large isolated immunoreactive cells were present in the connective tissue. A similar pattern of Tβ_4_ expression was observed in the submandibular glands (Fig. 6b). Minor salivary glands at the base of the tongue were markedly immature, with branching tubuli containing immunoreactive granular deposits of the peptide (Fig. 6c), in the absence of a significant positivity in the loose mesenchyma.

**Figure 7:**
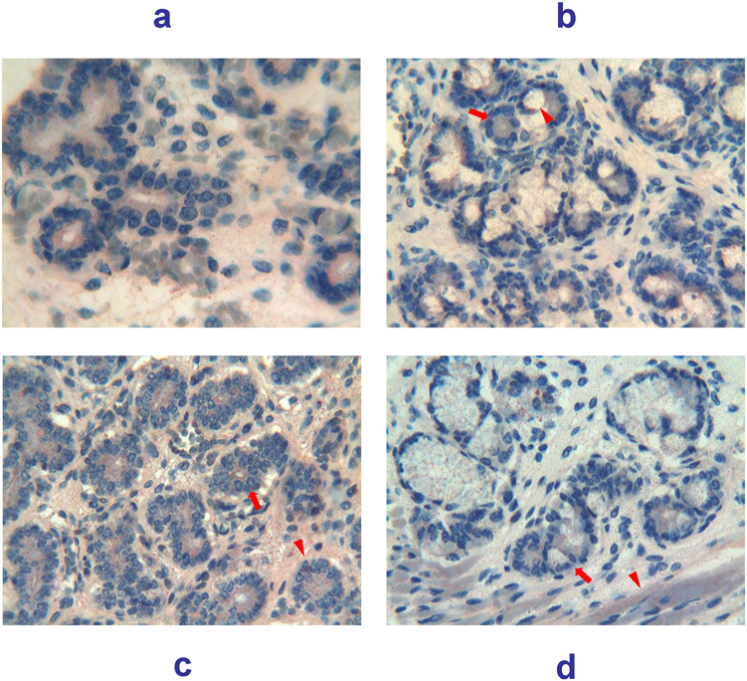
Immunohistochemical detection of thymosin β_4_ in salivary glands at 21 weeks (147 days) of gestational age. a) (400×) Parotid glands show a weak granular reactivity in the glandular cells, as well as in the loose mesenchyma. b) (250×) Submandibular glands show the presence of fine particles reactive for Tβ_4_ mainly stored in the cytoplasm of both serous (arrow) and mucous cells (arrowhead). c) (250×) Sublingual glands show large amounts of Tβ_4_ as fine and coarse granules in the cytoplasm of acinar (arrowhead) and ductal cells (arrow) and in the periglandular mesenchyma. d) (250×) Minor salivary glands showing large amounts of Tβ_4_ in the cytoplasm of acinar serous and mucous cells (arrow), as fine and coarse granules. Tβ_4_-positive granules are also observed in the mesenchyma and in muscle cells (arrowhead).

21 weeks (147 days of GA). The parotid showed reactivity in the glandular cells, appearing as granular deposits in the apical cytoplasm of cells as well as in the lumen (Fig. 7a). Submandibular glands exhibited, in this subject, a degree of maturation much higher when compared with the same glands of 20 weeks subject, in spite of a single week of difference in the gestational age. Acini appeared well differentiated with evident serous and mucous cells. Fine particles reactive for Tβ_4_ were mainly detected in the cytoplasm of both serous and mucous cells (Fig. 7b), whereas reactivity for the peptide in the connective tissue was very mild. In sublingual gland reactivity was seen in the apical cytoplasm of epithelial cells and in the lumen, whereas reactive granules were diffusely present in the connective tissue (Fig. 7c). Minor salivary glands were well developed for the gestational age, often showing a predominance of mucous cells in the acini. Tβ_4_ were present in the cytoplasm of acinar cells, as fine and coarse amounts (Fig. 7d). Tβ_4_ positive granules were also observed in the mesenchyma surrounding the epithelial structures. Muscle cells of the tongue showed a weak homogeneous reactivity for the peptide (Fig 7d).

**Figure 8:**
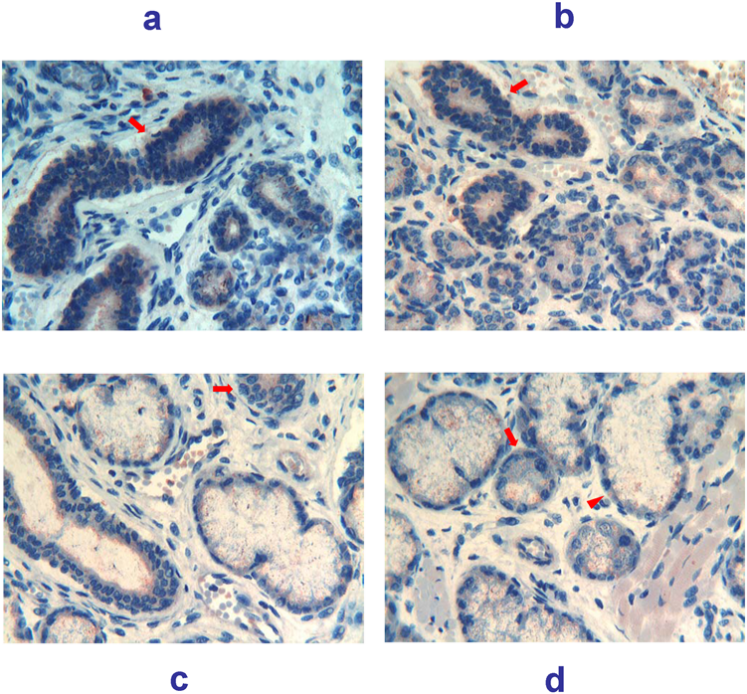
Immunohistochemical detection of thymosin β_4_ in salivary glands at 31 weeks (217 days) of gestational age. a) (250×) Parotid and b) (250×) submandibular glands showing a diffuse reactivity for Tβ_4_ localized in the cytoplasm of tubular cells (arrow). Some granular positivity is also present in the lumen. c) (250×) sublingual and d) (250×) minor salivary glands showing a lower degree of immunoreactivity, characterized by the presence of granules of different size in the cytoplasm of serous (arrow) and mucous cells (arrowhead).

31 weeks of gestation (217 days of GA). The pattern of reactivity for Tβ_4_ observed in the major salivary glands was significantly different from that observed in the previous stages. Parotid (Fig 8a) and submandibular (Fig 8b) glands showed a similar immunohistochemical pattern characterized by a diffuse reactivity for Tβ_4_ localized in the cytoplasm of tubular cells, associated with a granular positivity in the lumen. Sublingual (Fig. 8c) and minor (Fig. 8d) glands showed a lower degree of immunoreactivity, characterized by the presence of particles of different size in the cytoplasm of serous and mucous cells.

**Figure 9:**
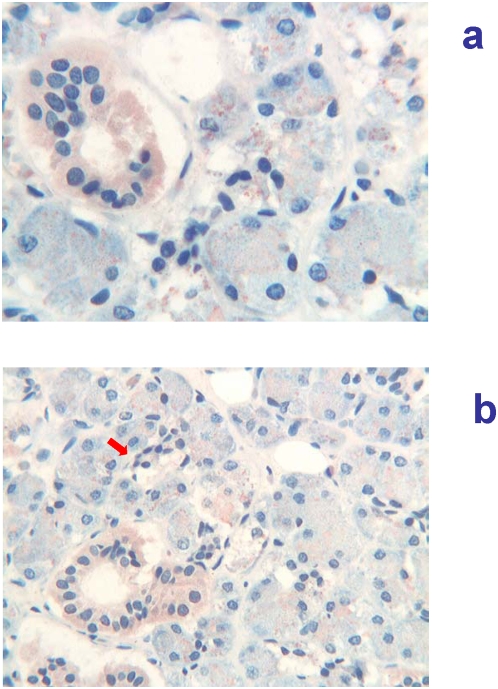
Immunohistochemical detection of thymosin β_4_ in salivary glands in an infant 18 month old. a) (400×) submandibular and b) (250×) sublingual glands showing the highest degree of immunoreactivity in duct cells. Coarse granular deposits of Tβ_4_ are also present in tubuloacinar cells (arrow).

1.5 year. In this subject only specimens from submandibular and sublingual glands were available (Figs. 9a,b). The pattern of reactivity for Tβ_4_ changed completely: in submandibular salivary glands, ducts showed the highest degree of immunoreactvity, which appeared as an homogeneous staining of the entire cytoplasm (Fig. 9a). Tβ_4_ was also detected in the cytoplasm of acinar cells, mainly as coarse granular deposits irregular in shape. A similar pattern of reactivity was observed in the sublingual gland acini and tubules (Fig. 9b).

**Figure 10:**
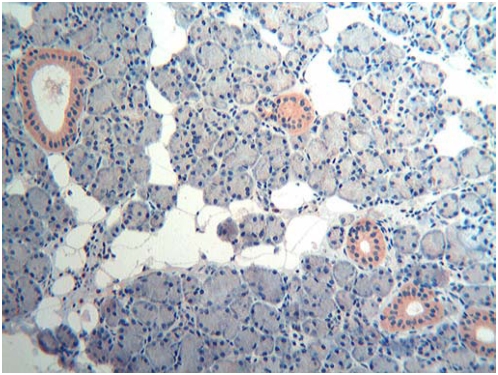
Immuno-histochemical detection of thymosin β_4_ in the parotid gland of an adult subject (100×). Tβ_4_ immunoreactivity is restricted to the cytoplasm of intra- and interlobular striated duct cells, appearing as homogeneous diffuse staining of the entire cytoplasm.

Adults. In major and minor salivary glands of the five adult subjects tested, utilized in this study as a control group, at variance with prenatal developmental stages, the pattern of reactivity for Tβ_4_ was characterized by the absence of granular deposits. Tβ_4_ detection was restricted to the cytoplasm of intra- and interlobular striated duct cells, as homogeneous diffuse staining of the entire cytoplasm. An example of parotid immunoreactivity is reported in Fig. 10.

## Discussion

Trends in the concentrations of Tβ_4_ and Tβ_10_ observed as a function of PMA (Figs. 1 and 2) lead us to suppose higher values in oral cavity before 190 days (27 weeks) of GA. On the obvious impossibility to extend the HPLC-MS analysis of whole saliva at lower GA ranges and with the aim at finding the sources responsible for the high concentration observed in the oral cavity of pre-term newborns, we addressed our experimental plan towards immunohistochemical detection of Tβ_4_. This plan was carried out utilizing salivary glands obtained by autopsy of fetuses and newborns of different gestational ages. This approach allowed to extend the detection of the potential Tβ_4_ sources till 90 days (12 weeks) of GA and to obtain information on the evolution of Tβ_4_ production and secretion during fetal growth.

The variability observed can partly derive from inter- and intra-individual variability of salivary flow rate. The measurement of this parameter was not possible in pre-term and at-term newborns for ethical reasons. However, on the whole, HPLC-MS and immunohistochemical data clearly indicate glandular expression of Tβ_4_ in fetuses inversely related to PMA with an apex of expression at about 140/150 days (20/21 weeks) of GA, differently from what observed in adults, where oral thymosins mainly derive from gingival crevicular fluid [13].

The actual un-availability of specific antibodies raised against Tβ_10_ (to the best of our knowledge) did not allow us to compare the expression of this peptide with respect to that of Tβ_4_. However, the increase in concentration of both determined by HPLC/MS analysis of oral fluid, suggested a trend for Tβ_10_ similar to that one observed of Tβ_4_ by immunochemistry. Some roles of the Tβ_4_ and Tβ_10_ couple are linked to a subtle modification of their relative amounts [11]. However, the change of concentration at constant peptide ratio (Fig. 2) observed in the present study suggests that the absolute thymosin quantities should play relevant functions during fetal development.

The major outcomes of morphological results suggest that immunochemical pattern of Tβ_4_ is dynamic and that it changes during the different stages of pre-natal and post-natal development. Particularly, a granular Tβ_4_ pattern predominates in the early and middle phases of fetal development, progressively substituted by a structural diffuse cytoplasmic staining as seen in full term infants and adults (Figs. 9 and 10). The granular pattern comprises both exocrine and paracrine features, the former were represented, at immunoistochemical level, by Tβ_4_-reactive granules inside the tubular lumen and in the apical cytoplasm of the acinar cells; the latter was represented by large peptide amounts in the mesenchymal space, as evident from Figs. 6a and 6b that may suggest a role of Tβ_4_ in the control of salivary gland branching morphogenesis. On the other hand, the pattern of thymosin we observed in histochemical preparation reflects the glandular differentiation and namely the start of acinar cell differentiation. Such a change is reflected even in development of tight junctions as demonstrated by immunohistochemical studies of claudins [19].

The presence of high amounts of thymosins β_4_ and β_10_ in the oral cavity of pre-term infants with a probable maximum of expression around 140–150 days (20–21 weeks, Figs. 6,7a–d), starting at least from about 85 days (12 weeks) of GA (Figs. 4a–c), put forward a role for these peptides in the development of oral cavity and its annexes. Until now the salivary glands were believed organs of slow development, reaching a complete secretory maturation after birth. Surprisingly, our results suggest that salivary glands do possess specific secretory capability even during prenatal maturation stages. In fact, at least since 85 days (12 weeks) of GA they start secreting peptides, such as thymosins β_4_ and β_10_, which should have specific roles during development. After an apex reached at around 150 days (21 weeks) of GA, production of these peptides decreases rapidly and the secretion of salivary glands slowly switches towards the expression and secretion of salivary peptides typical of adult age (i.e. acidic proline-rich proteins [15], statherin, histatins, basic proline-rich proteins and salivary cystatins). The switch of secretion disclosed by HPLC-MS analysis of whole saliva, was confirmed by immunoistochemical analysis of developing salivary glands, which evidenced a progressive decrease in the secretion of Tβ_4_ starting from the middle phases of foetal development. The switch could involve other exocrine and endocrine glands during foetal development. This study offers only a partial description of this switching phenomenon and obviously it does not pretend to establish any role for this high Tβ_4_ and Tβ_10_ salivary gland production during a particular period of foetal life, even though the immunohistochemical results are suggestive for a contemporaneous paracrine and exocrine functions. The exocrine pathway arises puzzling questions about the environment where the peptides are potentially released. In fact, salivary glands discharge during foetal life directly in amniotic fluid, where thymosins may be dispersed. The secretion of Tβ_4_ and Tβ_10_ from salivary gland might be connected to a demand of the whole amniotic sac. On the other hand, fluid secreted by salivary glands may be ingested and addressed towards the foetal gastrointestinal and respiratory tracts putatively influencing their pre-natal development. Alternatively, potential interactions with specific mucins could maintain these peptides adherent to the epithelium of the oral cavity.
